# Exploring the Comorbidity Mechanism of Internet Addiction, Insomnia, Depression, and Suicidality Among Chinese College Students Through Network Analysis

**DOI:** 10.31083/AP46397

**Published:** 2026-08-06

**Authors:** Huayu Li, Hongyuan Lv, Jiahui Zhang, Yu Fu, Jianpeng Zhang, Hongmei Wang

**Affiliations:** ^1^Department of Social Medicine of School of Public Health and Department of Pharmacy of the First Affiliated Hospital, Zhejiang University School of Medicine, 310058 Hangzhou, Zhejiang, China; ^2^School of Physical Education of Yantai University, 264005 Yantai, Shandong, China

**Keywords:** students, internet addiction, insomnia, depression, suicidal ideation, network analysis

## Abstract

**Background:**

Internet addiction is prevalent among college students and is linked to adverse mental health outcomes such as insomnia, depression, and suicidality. However, the variability in internet addiction patterns among college students and its connection to mental disorders remains insufficiently explored. This study examined the comorbidity network of internet addiction, insomnia, depression, and suicidality in Chinese college students through latent profile analysis (LPA) and network analysis (NA).

**Methods:**

Data from 3127 Chinese college students were collected using the Internet Addiction Test (IAT), Insomnia Severity Index (ISI), Patient Health Questionnaire-9 (PHQ-9), and Suicidal Behavior Questionnaire-Revised (SBQ-R). LPA was applied to identify subgroups with similar internet addiction profiles. NA was conducted among addicted users to map symptom associations, and network comparison tests (NCT) were used to evaluate subgroup differences.

**Results:**

Three distinct user profiles emerged: regular users, moderate users, and addicted users. NA revealed that the central symptom of internet addiction was “lack of self-control when online”. Furthermore, “trouble sleeping”, “frequency of suicidal ideation over the past year” and “sleep maintenance (middle)” were identified as bridge symptoms, connecting insomnia, depression, and suicidality with internet addiction. NCT indicated no significant gender differences in overall network strength.

**Conclusions:**

Interventions focused on improving self-regulation and addressing sleep problems should be prioritized to help reduce internet addiction, insomnia, depression, and suicidality in this population.

## Main Points

1. This study identified three distinct profiles of internet use among college students: regular users, moderate users, and addicted users, with the addicted user profile exhibiting the most severe symptoms of insomnia, depression, and suicidality.

2. Network analysis revealed that “lack of self-control when being online” is a central symptom of internet addiction, while “trouble sleeping”, “frequency of suicidal ideation in the past year”, and “sleep maintenance (middle)” act as key bridge symptoms connecting internet addiction to mental disorders.

3. These findings highlight the need for targeted interventions that address both self-regulation skills and sleep hygiene, and provide psychological support to reduce suicidal risks, in order to effectively prevent and treat internet addiction and its associated mental health comorbidities among college students.

## 1. Introduction

Over the past decade, technological advancements have made the internet an integral part of daily life, with the global number of Internet users reaching 4.9 billion in 2021, accounting for more than two-thirds of the world’s population [1]. Since its inception, the internet has offered numerous advantages, including enhanced social connectivity, active engagement, and expanded access to information. However, excessive internet use has detrimental effects on health, contributing to issues such as social anxiety, depression, low self-esteem, poor sleep quality, and heightened stress levels [2]. Internet addiction refers to the compulsive and excessive use of the internet, characterized by withdrawal symptoms, a persistent urge to engage in online activities, and other related symptoms [3,4]. It is important to note that internet addiction is a multidimensional construct which includes several subtypes, such as internet gaming disorder (IGD), with potentially different neurobiological underpinnings and clinical manifestations [5]. Adolescents, particularly college students, often find themselves in an environment with relatively few restrictions and may lack the necessary self-control, which makes them more vulnerable to developing internet addiction [6]. This heightened vulnerability can be linked to a combination of neurobiological and social influences. For example, many parents provide their children with internet-connected devices during their first year of college to help them stay in touch, offering them easy access to online platforms [7]. Furthermore, the reduced supervision from educators and parents grants university freshmen greater independence in their internet usage. As students transition from a structured academic environment to one that emphasizes self-directed learning, they experience increased flexibility in managing their online time. This stage of life offers more convenient and unrestricted access to the internet, which amplifies the likelihood of developing internet addiction, particularly among those with lower levels of self-control. Additionally, the boarding school setting shifts communication away from familial interactions, placing more emphasis on peer relationships, while daily life demands increased self-sufficiency and collaboration with others. These changes often lead to heightened social interaction needs. Students often avoid face-to-face interactions due to social anxiety [8]. For these individuals, online communication that does not require direct personal contact provides a more comfortable environment. However, this preference can lead to the development of maladaptive thought patterns, which in turn may increase the risk of internet addiction. The prevalence of internet addiction differs across regions and cultures, largely due to variations in diagnostic criteria and the assessment tools used. A comparative analysis of internet addiction rates in various countries reveals a higher prevalence in certain Asian nations compared to the United States. Data from a study of 8067 college students aged 18–30 across six Asian countries show that the rates of internet addiction were 12.9% in Japan, 13.8% in China, and 9.3% in Singapore, in contrast to the 8% observed among students in the United States. Additionally, Asian students diagnosed with internet addiction were found to be more susceptible to depression compared to their American counterparts [9]. Although internet addiction is not yet recognized by the Diagnostic and Statistical Manual of Mental Disorders (DSM), it has become a major challenge and public health problem in China nowadays and is receiving increasing attention from psychiatrists and educators. A significant amount of research has demonstrated that internet addiction among college students is strongly linked to a range of mental health disorders, including both psychopathological symptoms and personality disorders [10]. For instance, prior studies have found that internet addiction was associated with conditions such as insomnia [11] depression [12], even suicidality [13]. Of particular relevance, emerging case studies highlight a potential link between severe forms like IGD and early onset psychosis in vulnerable young individuals, underscoring the serious mental health risks associated with problematic internet use [5]. Specifically, research indicates that college students with internet addiction experience notably poorer sleep quality and reduced sleep duration [14]. A meta-analysis revealed that individuals with internet addiction were 2.2 times more likely to suffer from insomnia compared to those without internet addiction and experienced significantly shorter sleep durations [2]. Furthermore, Li et al. [15] investigated the dose-response relationship between the amount of time spent online and depressive symptoms in children and adolescents, finding a significant association between internet addiction behaviors and an increased risk of depression. Meanwhile, research by Shen et al. [16] showed that individuals with internet addiction exhibit higher rates of suicidal behaviors, even after adjusting for confounding variables like depression. Prolonged engagement with the internet often occupies substantial time, reducing opportunities for face‑to‑face communication, adequate rest, and participation in daily routines [17], which may consequently elevate depressive symptoms [18]. Comorbidity, rather than a single mental health issue, often leads to a worse prognosis, greater dysfunction, and more interference in daily life [19]. Given that insomnia, depression, and suicidality are frequently associated with internet addiction, and these conditions often overlap, it is crucial to explore the independent relationships between internet addiction and other psychological issues. However, existing studies primarily examined the relationship between internet addiction and psychological problems by employing a variable-centered analytical method. This method assumes that all participants meet criteria for internet addiction, overlooking possible variability within the group. Such symptom heterogeneity may be masked, and individuals whose internet use is within normal limits could be misclassified, thereby diluting the observed associations between internet addiction and mental health outcomes. Moreover, the precise mechanisms linking internet addiction to depression, sleep disturbances, and suicidal behaviors remain insufficiently explored.

Latent profile analysis (LPA) is a statistical approach that focuses on identifying unobserved subgroups or latent profiles within data. The goal is to uncover distinct groups that exhibit different patterns or characteristics across the observed variables [20]. LPA is commonly applied to explore latent types or subgroups within a population, allowing for a deeper understanding of the data and the development of tailored interventions or treatment strategies [21]. When applied to internet addiction symptoms, this method can help identify various subgroups within the population. Network analysis (NA) is a relatively new technique rooted in dynamic systems modeling, offering novel perspectives on how individual symptoms interact within a broader network of variables [22]. In contrast to conventional frameworks, the network theory of mental disorders (NTMD) posits that symptoms are not merely consequences of an underlying disorder, but constitute the disorder itself as interconnected elements within the system [23]. This framework serves two primary functions. First, it identifies core symptoms by evaluating their relative significance within the disorder. Second, it explains comorbid conditions by examining symptoms that act as bridges, connecting different disorders. A symptom from one disorder can trigger symptoms in another, thereby playing a role in both the onset and continuation of comorbid conditions [23]. The combination of LPA and NA is widely used to explore the relationship between mental disorders and problematic internet use, particularly in the context of social media. For instance, research by Shan et al. [24] employed LPA to classify WeChat users into three distinct groups, with moderate and problematic users comprising 52.9% of the overall sample. By applying NA to the problematic users, the researchers found that excessive use of WeChat was linked to depressive symptoms through two bridging symptoms: sadness and pessimism. However, this study primarily focused on the connection between problematic WeChat use and depression, limiting its scope to a single platform and a specific mental health outcome. To the best of our knowledge, there is limited research that specifically examines the relationship between internet addiction and mental disorders such as insomnia, depression, and suicidality using a combination of LPA and NA. In this study, we begin by applying LPA to identify college students with internet addiction. Subsequently, NA is used to pinpoint the central symptoms of internet addiction within this group and to investigate how these symptoms influence psychological outcomes, including insomnia, depression, and suicidality.

## 2. Methods

### 2.1 Participants and Procedure

This study received ethical approval from the School of Public Health, Zhejiang University (Approval No. ZGL201312-1). It was conducted in compliance with the Helsinki Declaration. The participants in this study were freshmen, sophomores, and juniors from various academic disciplines at four universities in Shandong Province. We employed a multistage stratified cluster sampling method to maximize sample representativeness. First, four universities in Shandong Province were selected purposefully to cover different scales (small, medium, large) and disciplinary diversity (sciences, engineering, liberal arts, medicine). Within each university, the sample was stratified by academic year (freshman, sophomore, junior) and faculty type (e.g., science, engineering, liberal arts, medicine). For each stratum, intact classes were used as primary sampling units (PSUs), and classes were randomly selected proportionally to their enrollment size in that stratum. Because the sampling design ensured proportional allocation across strata, no sampling weights were applied. Nevertheless, given the hierarchical nature of the data (students nested within classes and universities), we used robust standard errors clustered at the class level in sensitivity analyses; these yielded results consistent with the main analyses, suggesting minimal impact of clustering on parameter estimates. The survey was distributed by class advisors using the Questionnaire Star platform (https://www.wjx.cn/) and shared within each class’s WeChat group. Students could access and complete the questionnaire by clicking the provided link. To ensure data quality, an attention-check item was embedded in the survey. Prior to participation, respondents were given comprehensive information about the study, informed that it was voluntary, and advised of their right to withdraw at any stage. Informed consent was obtained from all participants before data collection commenced.

### 2.2 Materials and Methods

#### 2.2.1 Internet Addiction Test (IAT)

The IAT developed by Young in 1998, is a widely used tool for assessing the prevalence and severity of internet addiction. It comprises 20 self-reported items, each rated on a six-point scale from 0 (“not applicable”) to 5 (“always”) [25]. The total score ranges from 0 to 100, with higher scores indicating a greater severity of internet addiction. The IAT evaluates six dimensions, each representing distinct symptom patterns: salience, excessive use, neglect of work, anticipation, lack of control, and neglect of social life [26]. A previous study has demonstrated that the IAT exhibits strong reliability and validity among college students [27]. In this study, the internal consistency coefficient was calculated to be 0.955.

#### 2.2.2 Insomnia Severity Index (ISI)

The presence and severity of insomnia were assessed using the ISI, a 7-item self-report measure designed to evaluate the nature, severity, and impact of insomnia experienced in the past month. Each item is rated on a 5-point Likert scale, ranging from 0 (no problem) to 4 (very severe problem), with total scores ranging from 0 to 28. The ISI score is categorized as follows: no insomnia (0–7), sub-threshold insomnia (8–14), moderate insomnia (15–21), and severe insomnia (22–28) [28]. In this study, the Cronbach’s α coefficient for the ISI was found to be 0.881.

#### 2.2.3 Patient Health Questionnaire-9 (PHQ-9)

Depressive symptoms were assessed using the PHQ-9 [29], which is based on the nine criteria for major depressive disorder as outlined in the DSM-IV. Participants responded to each item using a 4-point Likert scale, with options ranging from 0 (not at all) to 3 (most of the time or always), resulting in a total score between 0 and 27. Higher scores reflect more severe depression, with scores ≥5 indicating at least mild depressive symptoms; this value is applied as a screening threshold rather than a diagnostic boundary. The PHQ-9 has been extensively validated within the Chinese population and is regarded as a reliable measure for assessing depressive symptoms [30]. In this study, the internal consistency coefficient was 0.904.

#### 2.2.4 Suicidal Behavior Questionnaire-Revised (SBQ-R)

Suicide risk factors were assessed using the Suicidal Behaviors Questionnaire-Revised (SBQ-R) [31], a self-report instrument designed to evaluate: (1) the history of suicidal thoughts and attempts, (2) the frequency of suicidal ideation in the past year, (3) the communication of suicidal intentions, and (4) the individual’s self-perceived likelihood of future suicidal behavior. The scores across all items are summed, with total scores ranging from 3 to 18. Based on prior research in nonclinical populations, a score ≥7 was used as a risk indicator for elevated suicide-related behaviors. This is not equivalent to a clinical diagnosis, but rather reflects increased risk in epidemiological screening. The SBQ-R is a widely used tool, proven to have robust reliability and validity in assessing suicidal ideation and behaviors among students [32]. In this study, the internal consistency was deemed acceptable, with a Cronbach’s α of 0.767.

### 2.3 Data Analysis

Descriptive statistics were generated using IBM SPSS 27.0 (IBM Corp., Armonk, NY, USA). Categorical variables are presented as frequencies (N) and percentages (%), while continuous variables are expressed as means and standard deviations. Associations between variables were assessed using Spearman’s correlation analysis.

To identify distinct subgroups of internet addiction among college students, LPA was performed with Mplus 8.4 (Muthén & Muthén, Los Angeles, CA, USA). This person-centered method classifies individuals into latent profiles based on shared patterns across observed variables, offering an ecologically valid representation of real-world heterogeneity [33]. The 20 items of the IAT served as indicators. Models with an increasing number of profiles (starting from one) were evaluated. Model selection was guided by the Akaike Information Criterion (AIC), Bayesian Information Criterion (BIC), and sample size-adjusted BIC (aBIC), with lower values indicating better fit. The Lo-Mendell-Rubin likelihood-ratio test (LMRT) and the Bootstrapping likelihood-ratio test (BLRT) were used to determine the optimal number of profiles; a significant *p*-value (<0.05) suggests that a model with k profiles fits better than a model with k-1 profiles. Classification accuracy was assessed via entropy (values closer to 1 indicate better separation) and average posterior probabilities (AvePP).

NA was implemented in R 4.4.2 (R Foundation for Statistical Computing, Vienna, Austria). We first visualized partial‑correlation networks using qgraph with a spring layout, treating internet‑addiction items (or dimensions) and mental‑health items as separate communities. To obtain sparse and interpretable graphs, edges were estimated with EBICglasso (γ = 0.5) after computing polychoric or Pearson correlations via cor_auto, depending on measurement level [34]. Traditional centrality indices (especially strength) were derived, while closeness and betweenness were interpreted cautiously due to known instability in psychological networks. Bridge metrics were computed using the networktools package (bridge strength, bridge betweenness, and bridge closeness) to locate nodes linking different symptom communities [35].

We assessed accuracy and stability with bootnet (Version 1.5.0, https://cran.r-project.org/package=bootnet). Precision of edge estimates was examined via non‑parametric bootstraps (1000 resamples; 8 cores) to obtain 95% CIs. Robustness of centrality estimates was evaluated using case‑dropping subset bootstrap; the correlation‑stability (CS) coefficient was reported, with ≥0.25 considered acceptable and >0.50 preferred [36]. We also ran bootstrapped difference tests to compare edges and node strengths. Between‑group differences in network structure and global strength were examined with the Network Comparison Test (NCT) [37]. Finally, a multivariate generalized estimating equations (GEE) model was fitted with the IAT total score as the dependent variable. Independent variables included latent profile membership, insomnia, depression, and suicidality scores, with adjustments for demographic and health behavior covariates.

## 3. Results

### 3.1 Demographic Characteristics of Participants

A total of 4037 college students were invited to participate in the study. To maintain the quality of responses, one attention check item was included in the survey. Additionally, participants who completed the survey in under 150s were excluded from the analysis. As a result, 910 participants were removed from the dataset, yielding a final response rate of 77.5%. Missing data were handled using a complete-case analysis (listwise deletion). The percentage of missing data for each measure was minimal (less than 2%). As shown in **Supplementary Table 1**, there were no significant demographic differences between participants with complete data and those with missing data. Participants provided demographic information, including age, gender, race, residence, family sibling status, body mass index (BMI), smoking and alcohol use, physical activity, dietary habits, and levels of internet addiction, insomnia, depression, and suicidality. The study ultimately included 3127 valid responses from college students, with a mean age of 19.47 years (ranging from 16 to 24). Among the participants, 45.1% were male and 54.9% were female. Comprehensive demographic data are presented in Table  1. The correlation analysis indicated significant positive relationships between internet addiction, insomnia, depression, and suicidality (**Supplementary Fig. 1**).

**Table 1.** **Depiction of participant demographic information**.

Variable		Total (n = 3127)	Regular users (n = 1577)	Moderate users (n = 1100)	Addicted users (n = 450)	χ^2^/*F*	*p*
Gender						122.531	<0.001
	Male	1410 (45.1)	865 (54.9)^a^	390 (35.5)^b^	155 (34.4)^b^		
	Female	1717 (54.9)	712 (45.1)^a^	710 (64.5)^b^	295 (65.6)^b^		
Race						0.601	0.740
	Han	2989 (95.6)	1503 (95.3)^a^	1055 (95.9)^a^	431 (95.8)^a^		
	Other	138 (4.4)	74 (4.7)^a^	45 (4.1)^a^	19 (4.2)^a^		
Residence						5.076	0.079
	Urban	1042 (33.3)	554 (35.1)^a^	341 (31.0)^a^	147 (32.7)^a^		
	Rural	2085 (66.7)	1023 (64.9)^a^	759 (69.0)^a^	303 (67.3)^a^		
Family sibling status						15.056	<0.001
	Only child	929 (29.7)	516 (32.7)^a^	284 (25.8)^b^	129 (28.7)^a,b^		
	Non-only child	2198 (70.3)	1061 (67.3)^a^	816 (74.2)^b^	321 (71.3)^a,b^		
BMI						8.301	0.081
	Low weight	471 (15.1)	210 (13.3)^a^	180 (16.4)^a,b^	81 (18.0)^b^		
	Normal	1945 (62.2)	1000 (63.4)^a^	676 (61.5)^a^	269 (59.8)^a^		
	Overweight/obesity	711 (22.7)	367 (23.3)^a^	244 (22.2)^a^	100 (22.2)^a^		
Smoking status						13.565	<0.001
	Yes	225 (7.2)	140 (8.9)^a^	59 (5.4)^b^	26 (5.8)^a,b^		
	No	2902 (92.8)	1437 (91.1)^a^	1041 (94.6)^b^	424 (94.2)^a,b^		
Alcohol status						4.071	0.131
	Yes	618 (19.8)	334 (21.2)^a^	200 (18.2)^a^	84 (18.7)^a^		
	No	2509 (80.2)	1243 (78.8)^a^	900 (81.8)^a^	366 (81.3)^a^		
Physical activity						156.770	<0.001
	Low	171 (5.5)	64 (4.1)^a^	58 (5.3)^a^	49 (10.9)^b^		
	Moderate	2037 (65.1)	905 (57.4)^a^	797 (72.5)^b^	335 (74.4)^b^		
	High	919 (29.4)	608 (38.6)^a^	245 (22.3)^b^	66 (14.7)^c^		
Regularity of meals						90.965	<0.001
	Irregular	586 (18.7)	245 (15.5)^a^	197 (17.9)^a^	144 (32.0)^b^		
	Generally regular	2165 (69.2)	1088 (69.0)^a^	799 (72.6)^a^	278 (61.8)^b^		
	Regular	376 (12.0)	244 (15.5)^a^	104 (9.5)^b^	28 (6.2)^b^		
ISI score		4.66 ± 4.38	3.02 ± 3.57^a^	5.58 ± 3.87^b^	8.16 ± 5.31^c^	337.719	<0.001
PHQ-9 score		5.56 ± 4.87	3.39 ± 3.91^a^	6.68 ± 4.04^b^	10.44 ± 5.29^c^	559.697	<0.001
SBQ-R score		4.13 ± 2.06	3.49 ± 1.32^a^	4.46 ± 2.14^b^	5.58 ± 2.89^c^	233.044	<0.001

Note: BMI, body mass index; ISI, Insomnia severity index; PHQ-9, Patient health questionnaire-9; SBQ-R, Suicidal behavior questionnaire-revised. ^a^, ^b^, and ^c^ indicate homogeneous subgroups determined by post-hoc pairwise comparisons after Bonferroni adjustment. Categories sharing the same letter are not significantly different at the 0.05 level (adjusted *p*-value). Unadjusted *p*-values are shown in the *p* column.

### 3.2 LPA for College Students

In this study, the 20 items of IAT were used as observed variables, and potential profile models with 1 to 5 profiles were tested. The fit indices for these models are shown in Table 2. With the addition of more profiles, the fit indices (AIC, BIC, and aBIC) showed a continuous decline, indicating better model fit. Nevertheless, in the four- and five-profile solutions, the smallest group comprised less than 5% of the sample. Balancing statistical fit with practical relevance, the three-profile model was identified as the most suitable representation of the data. This model yields an entropy value of 0.957, which reflects a high level of classification accuracy. Furthermore, we validated the classification quality by calculating the AvePP: the AvePP values for the three classes were 0.971, 0.987, and 0.987, respectively, with corresponding misclassification rates all below 3%, supporting the reliability of the classification. Model stability analysis revealed that under the setting of 200 random starts and 10 final optimizations, the best log-likelihood value (–150,910.870) was consistently replicated across multiple runs, ruling out issues of local maxima.

**Table 2.** **Model fit indices for profile solutions**.

Number of profiles	AIC	BIC	aBIC	Entropy	LMRT	BLRT	Proportion
1	187,022.324	187,264.237	187,137.141	-	-	-	-
2	159,831.788	160,200.705	160,006.883	0.963	<0.001	<0.001	2132/995
3	150,910.870	151,406.792	151,146.244	0.957	<0.001	<0.001	1577/1100/450
4	147,519.687	148,142.613	147,815.340	0.944	0.176	<0.001	1406/604/967/150
5	145,728.288	146,478.218	146,084.219	0.930	0.084	<0.001	860/1281/566/352/68

AIC, Akaike Information Criterion; BIC, Bayesian Information Criterion; aBIC, sample size-adjusted BIC; LMRT, Lo-Mendell-Rubin likelihood-ratio test; BLRT, Bootstrapping likelihood-ratio test.

**Fig. 1.**
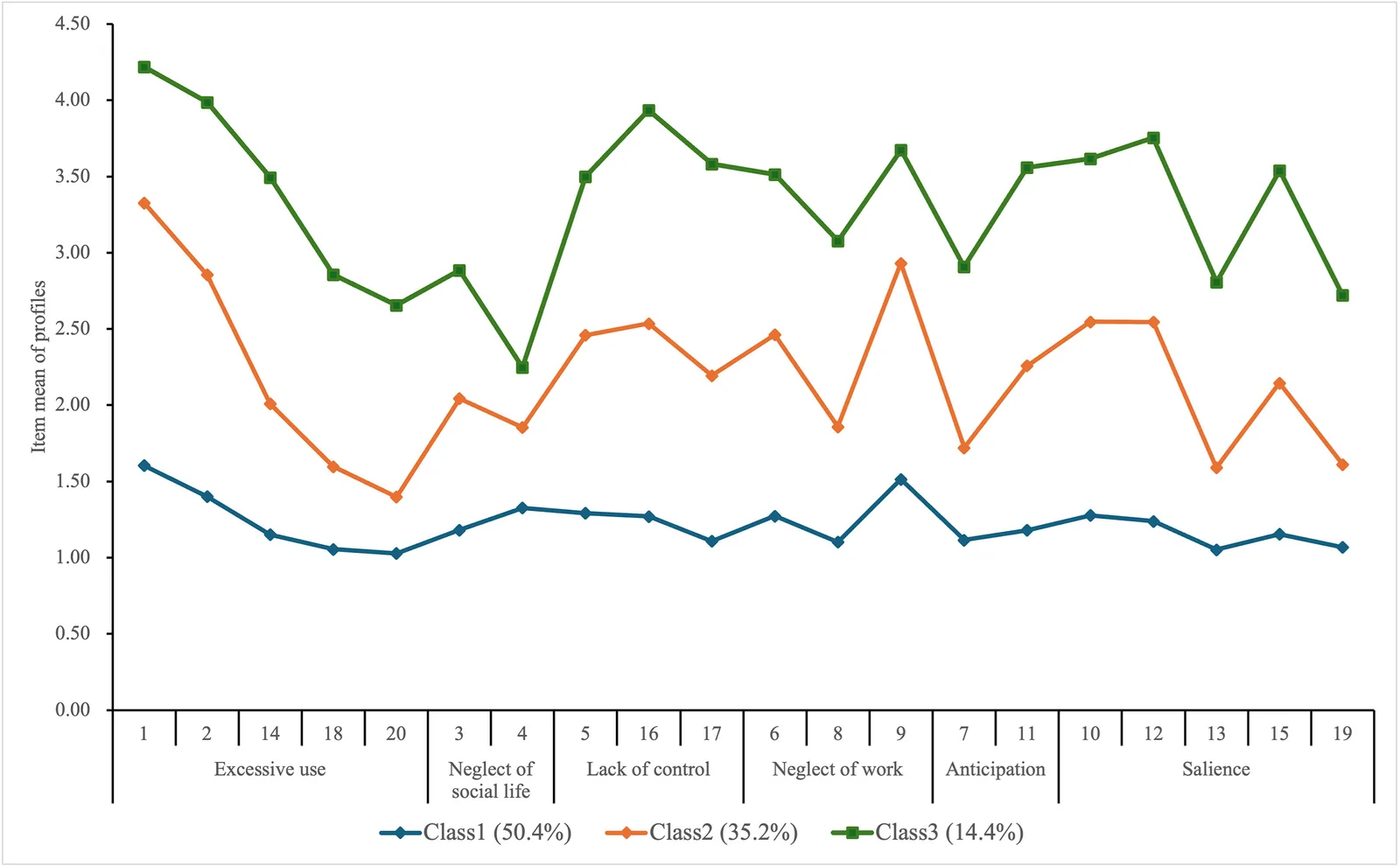
**The scoring characteristics of three profiles in 20-item behaviors of internet addiction**.

As depicted in Fig. 1, the first profile, comprising 50.4% of students, shows the lowest average scores on the 20 items and is termed the “regular users”. The second profile, representing 35.2% of students, is characterized by moderate scores across all 20 items of internet addiction and is labeled the “moderate users”. The third profile, consisting of 14.4% of students, exhibits the highest scores, indicating a higher risk of problematic internet use, and is referred to as the “addicted users”. We provide detailed fit index trend plots (**Supplementary Fig. 2**) and profile mean plots (**Supplementary Fig. 3**) in the supplementary materials.

### 3.3 Cross-Class Comparisons

We examined sociodemographic differences among the three groups. Statistically significant variations were found in gender (χ^2^ = 122.531, *p* < 0.001), family sibling status (χ^2^ = 15.056, *p* < 0.001), smoking status (χ^2^ = 13.565, *p* < 0.001), physical activity (χ^2^ = 156.770, *p* < 0.001) and regularity of meals (χ^2^ = 90.965, *p* < 0.001). To explore differences in insomnia, depression, and suicidality across the groups, we performed ANOVAs on the total scores of the ISI, PHQ-9, and SBQ-R, with the results shown in Table 1. Significant differences in the total scores of the three scales emerged across three classes. *Post hoc* comparisons revealed that the “addicted users” (class 3) had notably higher scores for insomnia, depression, and suicidality (all *p* < 0.001).

### 3.4 Network Analysis of Internet Addiction

**Fig. 2.**
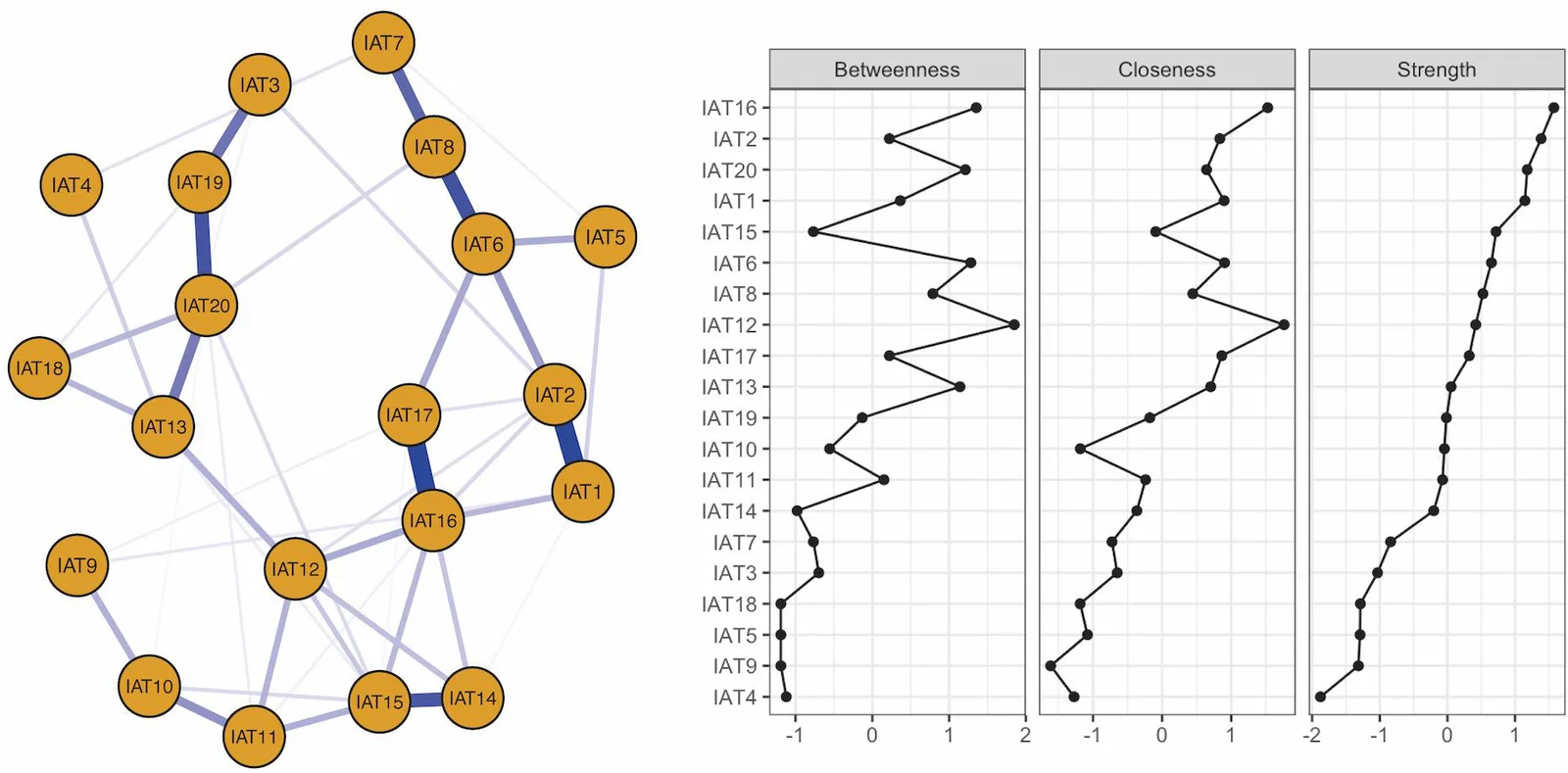
**Network structure of internet addiction**. Blue edges constitute positive partial correlations between variables. The edge thickness represents the strength of the association between symptom nodes. Centrality (Z score) of network was shown in right panel. IAT, Internet Addiction Test.

Fig. 2 presented the network diagram illustrating the conditional associations among the 20 items of the IAT scale for the “addicted users” group. Each circle in the diagram represents a node corresponding to an item, and the edges indicate the strength of the associations between these nodes. The meaning of each node abbreviation is explained in **Supplementary Table 2**. The edge linking node IAT1 “stay online longer than intended” and node IAT2 “neglect household chores” has the strongest association (edge weight = 0.416). Node IAT16 (“lack of self-control when online”) exhibited the highest strength centrality, indicating a strong positive association with other symptoms and positioning it as a key symptom within the network. Node IAT12 (“life is boring without the internet”) exhibited the highest closeness, indicating its significant role in linking otherwise unrelated symptoms within the network. Additionally, node IAT12 ranked highest in betweenness centrality, highlighting its function as a key mediator, connecting different symptoms and acting as a bridge within the network. The strength values for all nodes in the network was displayed in **Supplementary Fig. 4**, and no statistical difference was discovered for strength between the nodes IAT16 and IAT2. The edge stability assessment showed minimal overlap, suggesting that the network demonstrated high stability (**Supplementary Fig. 5**). The centrality stability was preferable in this network, as the CS coefficients for strength was 0.673 (**Supplementary Fig. 6**).

### 3.5 Network Analysis of Internet Addiction and Mental Disorders

**Fig. 3.**
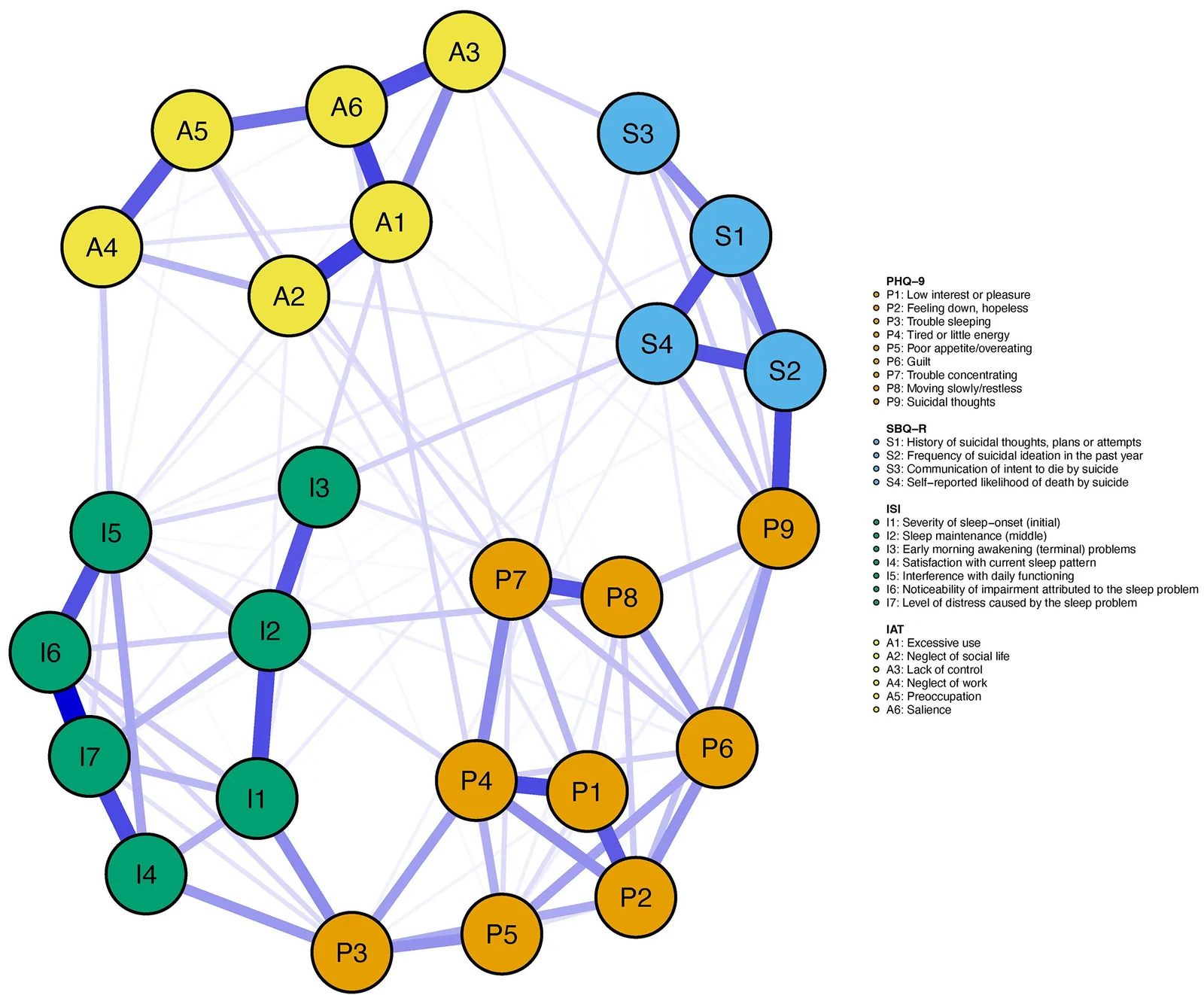
**Network structure of internet addiction and mental disorders**. Blue edges constitute positive partial correlations between variables. The edge thickness represents the strength of the association between symptom nodes.

Because the comorbidity network combining internet‑addiction and mental‑health symptoms would include around 40 nodes, exceeding typical recommendations for sample sizes near 250–350 for networks of this complexity [38], and our addicted‑user subgroup comprised 450 participants, we prioritized model parsimony. Specifically, rather than entering all 20 IAT items, we aggregated them into the instrument’s six commonly used dimensions (salience, excessive use, neglect of work, anticipation, lack of control, and neglect of social life) and used these as the internet‑addiction community. The resulting network structure for internet addiction, insomnia, depression, and suicidality was illustrated in Fig. 3.

Within the insomnia community, the strongest edge connected I6 (“noticeability of impairment”) and I7 (“distress caused by sleep problems”) (edge weight = 0.408). In the depression community, the strongest link was between P1 (“anhedonia”) and P4 (“fatigue”) (edge weight = 0.288). For suicidality, the strongest connection was between S2 (“frequency of past-year suicidal ideation”) and S4 (“self-perceived likelihood of suicide”) (edge weight = 0.273). The strongest cross-community edges were between S2 (suicidality) and P9 (“suicidal thoughts”) (edge weight = 0.289), followed by node I1 “severity of sleep–onset (initial)” and node P3 “trouble sleeping” (edge weight = 0.182).

**Fig. 4.**
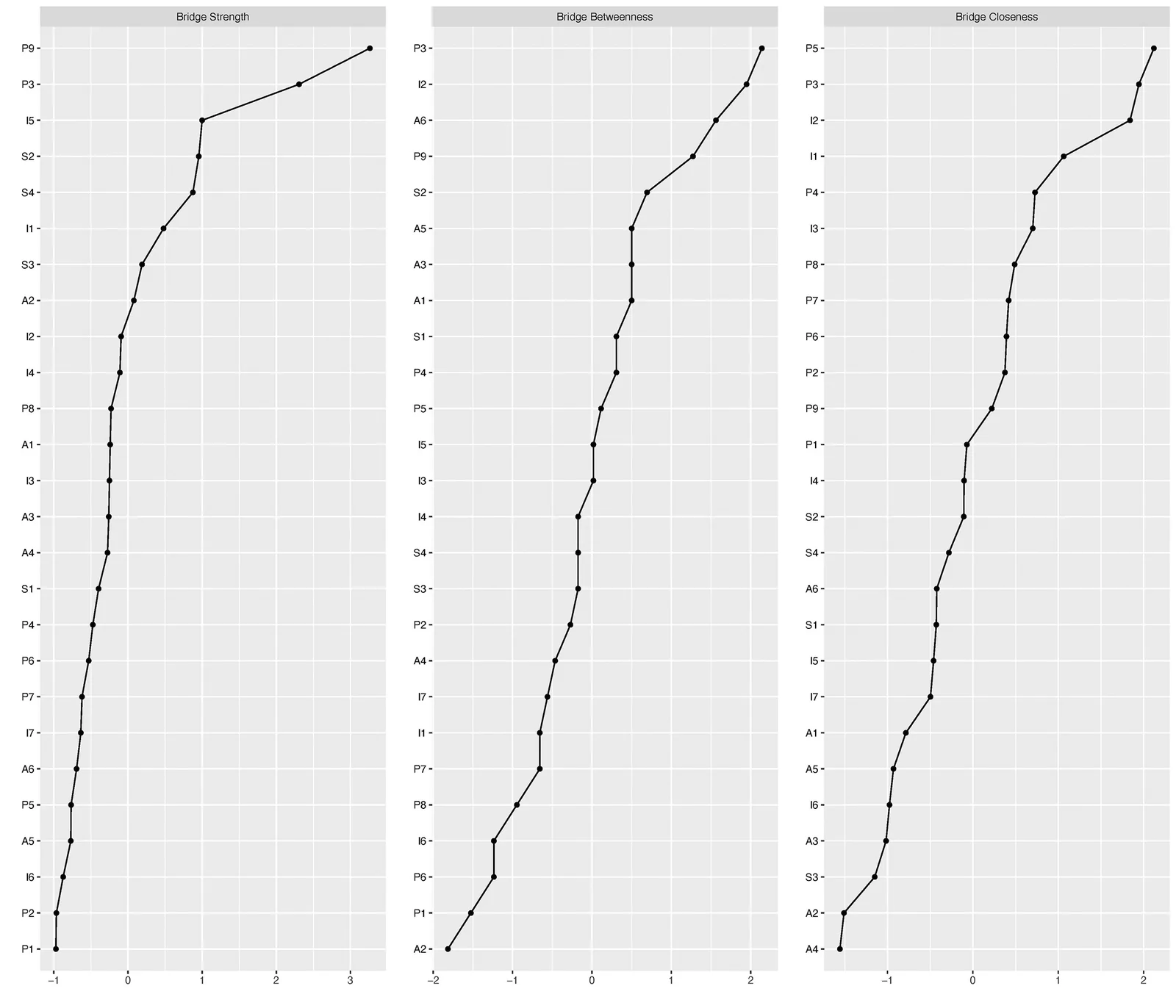
**Bridge centrality indices of the network between internet addiction and mental disorders**.

Centrality analysis (**Supplementary Fig. 7**) identified P4 (“fatigue”) and I2 (“sleep maintenance difficulty”) as the most central nodes in the overall network, based on their high rankings across strength, betweenness, and closeness indices. A difference test for strength indicated no significant difference between these two nodes (**Supplementary Fig. 8**). Bridge centrality analysis (Fig. 4) pinpointed key symptoms connecting the mental disorder communities to internet addiction. Specifically, P3 (“trouble sleeping”) was the primary bridge node from depression, S2 (“frequency of past-year suicidal ideation”) from suicidality, and I2 (“sleep maintenance difficulty”) from insomnia. Thus, P3, S2, and I2 were identified as critical bridge symptoms linking mental disorders to internet addiction. Difference tests for bridge strength are detailed in **Supplementary Fig. 9**.

**Supplementary Fig. 10** illustrated that the edge weights in the current sample were consistent with those in the bootstrapped samples and the bootstrapped 95% CIs were narrow, indicating a good accuracy of the network model. Stability was also satisfactory, with CS coefficients for both traditional strength and bridge strength reaching 0.673 (**Supplementary Figs. 11**,**12**), exceeding the recommended threshold of 0.5.

### 3.6 Network Comparison

We conducted a NCT to further explore the symptom characteristics of networks among college students with varying profiles of internet addiction. Significant differences in the global strength of the internet addiction networks were observed across the three groups, with values of 10.391 for “regular users” (class 1), 11.627 for “moderate users” (class 2), and 11.148 for “addicted users” (class3) (class 1 vs. class 2, *p* = 0.010; class 2 vs. class 3, *p* = 0.208; class 1 vs. class 3, *p* = 0.010). Additionally, we compared the symptom networks between subgroups of male (n = 155) and female (n = 295) students, as well as between only child (n = 129) and non-only child (n = 321). No significant differences in global network strength were found between these groups (both *p* > 0.05). It is worth noting that only covariates with sample sizes greater than 100 were included in the analysis to ensure sufficient statistical power.

### 3.7 Multivariate GEE Analysis

To validate the latent profiles and complement the network analysis from a population-averaged perspective, we performed a GEE analysis with the IAT total score as the dependent variable. As presented in **Supplementary Table 3**, the LPA profile emerged as the strongest predictor of internet addiction severity after adjusting for demographics and health behaviors. Compared to the “Regular Users” profile, the “Moderate Users” had a significantly higher IAT score (β = 8.52, 95% CI: 6.89–10.15, *p* < 0.001), and the “Addicted Users” profile demonstrated the largest increase (β = 21.73, 95% CI: 19.24–24.22, *p* < 0.001). The clinical scale scores for insomnia, depression, and suicidality, all showed significant positive associations with the IAT score (all *p*-values < 0.001).

## 4. Discussion

To the best of our knowledge, this study is the first to explore the interrelationships of mental disorders symptoms with internet addiction among college students using a combination of LPA and NA. Based on their patterns of internet use, the students were classified into three distinct profiles including “regular users”, “moderate users”, and “addicted users”. Internet addiction and mental disorders symptoms differed significantly across the subgroups. The “addicted users” group exhibited the most pronounced symptoms of insomnia, depression, and suicidality. Additionally, the NA identified IAT 16 (“lack of self-control when being online”) as the central node of the addicted user profile, and P3 (“trouble sleeping”), S2 (“frequency of suicidal ideation in the past year”), and I2 (“sleep maintenance (middle)”) were bridge nodes linking depression, insomnia and suicidality symptoms with internet addiction. Identifying central and bridging symptoms enhances our understanding of the comorbidity between internet addiction and mental disorders. These symptoms could be recognized as potential critical indicators for early detection and targeted intervention, warranting further investigation in longitudinal and interventional studies.

Compared to previous studies, the present study reported a higher prevalence of internet addiction, and this discrepancy can be attributed to the fact that earlier studies predominantly focused on adolescents populations [39] and social media use [40] within a broader framework. In contrast, our study specifically targeted college students, which led to variations in the study population, measurement tools, and other influencing factors. The 3-class model of internet addiction observed in the Chinese college student population closely mirrored earlier findings from a large sample of Chinese adolescents [41], implying itself as an appropriate classification for understanding internet use among young Chinese individuals. In our research, although three profiles showed relatively parallel paths, their characteristics were indeed discriminant. The “regular user” profile, representing 50.4% of the participants, had one peak value in item 9 (“alert when asked about online”) and scored lower than the other two profiles in each item. The “moderate users” profile, comprising 35.2% of the participants, had higher scores on item 1 (“stay online longer than intended”) and item 9. The “addicted users” profile, comprising 14.4% of the participants, scored higher than the other profiles on each item. The differences observed among the three profiles suggest that internet addiction among college students is a heterogeneous phenomenon. Interestingly, we observed that “moderate users” spent considerable time online (item 1) and remained alert during their internet activities (item 9), yet they reported relatively low levels of negative emotions (item 20). This finding suggests that, despite their high levels of internet engagement, these individuals did not experience the usual negative effects commonly linked to excessive use, nor did they display more pronounced symptoms of insomnia, depression, or suicidality. This phenomenon could be explained by the evolving role of the internet as an indispensable part of daily life, leading to an increase in both the frequency and duration of its usage. Recent findings by Fournier and colleagues [42] align with our research, proposing that extended online hours and concerns about privacy may be secondary factors in internet addiction, rather than core components. Consequently, a greater emphasis should be placed on addressing the negative impacts of internet usage to avoid pathologizing internet engagement.

Core symptoms are the most impactful indicators of a disorder, as they have the ability to trigger other symptoms and contribute to the progression of mental health issues [43]. Overall, identifying these central symptoms is crucial for effectively directing clinical interventions [44]. Our study identified IAT16 (“lack of self-control when being online”) and IAT2 (“neglect household chores”) as central symptoms of internet addiction among college students. This contrasts with previous research on internet addiction in adolescents and young adults, which highlighted IAT6 (“grades or school work suffer”) and IAT8 (“job performance or productivity suffer”) as core symptoms [45]. This discrepancy may be attributed to developmental differences between populations. Compared to adolescents who are under stricter academic supervision, college students experience greater autonomy and different responsibilities, making self-regulation challenges (rather than direct academic consequences) more salient in manifesting internet addiction. College students generally have greater access to the internet and more free time compared to adolescents, particularly high school students. In some cases, internet use is even encouraged due to online courses and assignments. Furthermore, college students face less stringent supervision and academic pressure than high school students, and they are not burdened by the financial demands of work. Psychologically, the developmental stage of college students—characterized by a search for identity and the formation of meaningful relationships—may additionally distinguishes them from adolescents. Interestingly, lack of self-control and neglect household chores appeared to play a key role in the development of internet addiction of college students. Previous research has shown that individuals with poor time management and self-control skills often find the internet to be a compelling escape from their demanding routines [46]. Additionally, people suffering from internet addiction tend to exhibit low self-esteem, heightened feelings of loneliness, and inadequate social skills [47]. The anonymity offered by the internet facilitates easier social interaction, which can be particularly appealing for those with internet addiction, as their social needs are often unmet in real-life situations [48]. This reliance on the virtual world for social fulfillment can result in individuals neglecting their real-world interactions, leading to excessive internet use and procrastination. In line with the practical implications suggested by our network findings, Cognitive behavioral therapy (CBT) may prove effective for students struggling with internet addiction, as they often hold the belief that life without online activities will feel dull and unfulfilling, which contributes to their difficulty in controlling internet use. Previous research has demonstrated that CBT can help reduce excessive internet usage, enhance time management skills, and promote emotional stability [49].

The network analysis offered symptom-level insights into the link between internet addiction and mental disorders, identifying sleep-related issues (“trouble sleeping” and “sleep maintenance [middle]”) as bridging symptoms between the two. In line with prior findings [50], students with higher internet addiction scores reported shorter sleep duration. Drawing on the displacement hypothesis [51], excessive internet use may replace protective real-life activities such as sleep, resulting in reduced sleep time and poorer sleep quality, thereby increasing vulnerability to psychological symptoms. However, improving sleep maintenance can help mitigate the adverse effects of internet addiction. Notably, promoting an earlier bedtime could extend sleep duration by approximately half an hour per night [52]. This finding suggests that encouraging earlier bedtimes and allowing for longer recovery sleep on weekends might be effective strategies to counteract the negative impacts of excessive internet use [53]. Consequently, sleep-related issues represent critical and pragmatic intervention points for preventing the comorbidity progression. Practical applications could include integrating sleep hygiene education and cognitive-behavioral techniques for insomnia (CBT-I) into standard intervention programs for students with internet addiction. Improving “sleep maintenance”, for instance, could act as a protective barrier, potentially disrupting the pathway from internet addiction to depression and suicidality. This approach aligns with the principle of parsimony in intervention design, as targeting a few key bridge symptoms may yield broad benefits across the entire comorbidity network. Furthermore, the study identifies a specific symptom of suicidality-frequency of suicidal ideation in the past year, also as bridge between internet addiction and mental disorders. There are several possible explanations for the direct link between internet addiction and suicidal ideation. First, the anonymity provided by the internet increases the likelihood of individuals with internet addiction being exposed to suicidal content or experiences [54]. Repeated exposure to such material may lead individuals to imitate suicidal ideation in the real world. Second, the phenomenon of de-individuation, which is common in online interactions, can diminish self-awareness, reduce concerns about social judgment, and lower the threshold for engaging in harmful behaviors such as suicide [55]. In this environment, individuals may be less aware of or less sensitive to the negative consequences of suicidal actions due to changes in cognitive control processes [56]. Lastly, the factors contributing to pathological behavior often overlap. As mentioned above, poor sleep quality, a common issue in internet addiction, may also exacerbate suicidal ideation among affected students. Our finding highlights the potential role of internet addiction in exacerbating mental disorders by affecting individuals’ sleep quality and emotional states. Specifically, prolonged internet addiction can lead to insufficient or disrupted sleep, which in turn increases the risk of depression. Suicidal ideation, in particular, may reflect the emotional distress and psychological pressure experienced by individuals, suggesting that those with internet addiction may lack effective coping mechanisms when faced with real-life challenges [57].

While demographic analysis revealed significant gender distribution differences across user groups (χ^2^ = 122.531, *p* < 0.001), the network comparison test demonstrated no significant gender differences in global network strength among addicted users. The absence of gender differences in network structure might reflect that once students develop problematic internet use patterns, the core mechanisms (particularly “lack of self-control” as the central symptom) become similar across genders, despite potential differences in initial susceptibility or usage preferences.

### Strengths and Limitations

This study offers several notable strengths. Firstly, stratified random sampling ensured a representative college student sample, enhancing the reliability and generalizability of the findings. Secondly, the survey-based methodology enabled large-scale data collection, encompassing students from a range of academic disciplines. This approach broadens our understanding of the relationship between internet addiction and mental health disorders in this population. Thirdly, and most importantly, this study employs a novel, two-stage analytical approach that integrates LPA and NA. This methodological innovation addresses a key limitation of prior research. We first used LPA to move beyond treating the population as homogeneous, successfully identifying a distinct, high-risk subgroup of students with problematic internet use patterns. Subsequently, applying NA to this precise subgroup allowed us to deconstruct the comorbidity architecture at a symptom level. This unique combination enabled the identification of not only central symptoms within internet addiction but, more critically, the bridge symptoms that directly connect it to insomnia, depression, and suicidality. This provides a mechanistic explanation for comorbidity and highlights precise targets for future interventions. Additionally, the use of the IAT questionnaire, which is well-validated for assessing internet addiction in the daily lives of Chinese college students, proved advantageous. Finally, the study addresses a pressing contemporary issue by exploring the connections between internet addiction and mental health, providing valuable insights that contribute to the ongoing dialogue and inform the development of potential interventions.

Despite the strengths of this study, there are several limitations that should be addressed in future research. First, the cross-sectional design restricts the ability to draw conclusions about causal relationships between internet addiction and mental disorders. Longitudinal studies would be valuable in understanding how internet addiction may affect mental health over time. Additionally, the reliance on self-report data may introduce social desirability bias and common method bias. To reduce these limitations, future studies could incorporate objective measures alongside self-reports. Furthermore, our sample was recruited from four universities in a single city in Eastern China, which may limit the generalizability of the findings to other regions (e.g., Central or Western China) or types of institutions (e.g., vocational colleges). Future studies should include more geographically and institutionally diverse samples to enhance external validity. Lastly, our study examined internet addiction in a general context and did not account for platform-specific heterogeneity. Different online platforms (e.g., short-video, social media, gaming, learning) serve distinct functions and are driven by varied user motivations. Our generalized measure could not capture these nuances. Future research should, therefore, investigate problematic usage by collecting platform-specific metrics (e.g., duration, frequency, engagement patterns on specific apps) and underlying motivations to offer a more nuanced understanding of how different digital environments contribute to comorbidity mechanisms. Based on our findings, we plan to conduct a 12-month longitudinal follow-up study with a multi-center sample, employing Ecological Momentary Assessment (EMA) and in-depth interviews to empirically test the causal hypotheses of the network model and explore optimal intervention timing.

## 5. Conclusions

The study identified internet addiction symptoms in 14.4% of college students, with “lack of self-control when online” appearing as the most salient manifestation. To address this, interventions aimed at enhancing self-regulation, improving time management abilities, and offering psychological support could be effective strategies for both preventing and treating internet addiction. Additionally, behaviors associated with sleep and suicidal ideation serve as bridges between internet addiction and mental disorders. Interventions targeting sleep issues may help mitigate the negative effects of internet addiction, while providing psychological support to reduce suicidal risks could effectively break the harmful cycle between addiction and mental disorders. Therefore, future intervention strategies should focus on these bridging symptoms, offering more comprehensive support to address both internet addiction and its associated mental health issues.

## Disclosure

The paper is listed as, “Exploring the Comorbidity Mechanism of Internet Addiction, Insomnia, Depression, and Suicidality Among Chinese College Students Through Network Analysis” as a preprint on Research Square at: https://www.researchsquare.com/article/rs-7549655/v1.

## Data Availability

The data supporting the findings of this study are available from the corresponding authors upon reasonable request.
