# Supplementary material for: Exploring the Comorbidity Mechanism of Internet Addiction, Insomnia, Depression, and Suicidality Among Chinese College Students Through Network Analysis
Source: Alpha Psychiatry. 2026 Aug 6;27(4):46397. doi: 10.31083/AP46397 (PMC13540041; doi:10.31083/AP46397)
Supplement: Supplementary file 1 [file 2757-8038-27-4-46397-s1.zip › Supplementary Material.docx]

Exploring the comorbidity mechanism of internet addiction, insomnia, depression, and suicidality among Chinese college students through network analysis

**Supplementary Table 1.** Demographic characteristics of included and excluded participants.

| Variable | Included Participants  (*n*=3127) | Excluded Participants  (*n*=910) | *χ^2^/F* | *P* |
| --- | --- | --- | --- | --- |
| Gender |  |  | 0.006 | 0.938 |
| Male | 1410(45.1) | 409(44.9) |  |  |
| Female | 1717(54.9) | 501(55.1) |  |  |
| Race |  |  | 0.011 | 0.340 |
| Han | 2989(95.6) | 863(94.8) |  |  |
| Other | 138(4.4) | 47(5.2) |  |  |
| Residence |  |  | 2.328 | 0.127 |
| Urban | 1042(33.3) | 328(36.0) |  |  |
| Rural | 2085(66.7) | 582(64.0) |  |  |
| Family sibling status |  |  | 0.221 | 0.638 |
| Only child | 929(29.7) | 263(28.9) |  |  |
| Non-only child | 2198(70.3) | 647(71.1) |  |  |
| BMI |  |  | 4.363 | 0.113 |
| Low weight | 471(15.1) | 128(14.1) |  |  |
| Normal | 1945(62.2) | 545(59.9) |  |  |
| Overweight/obesity | 711(22.7) | 237(26.0) |  |  |
| Smoking status |  |  | 0.156 | 0.693 |
| Yes | 225(7.2) | 62(6.8 |  |  |
| No | 2902(92.8) | 848(93.2) |  |  |
| Alcohol status |  |  | 1.934 | 0.164 |
| Yes | 618(19.8) | 199(21.9) |  |  |
| No | 2509(80.2) | 711(78.1) |  |  |
| Physical activity |  |  | 1.054 | 0.590 |
| Low | 171(5.5) | 53(5.8) |  |  |
| Moderate | 2037(65.1) | 605(66.5) |  |  |
| High | 919(29.4) | 252(27.7) |  |  |
| Regularity of meals |  |  | 2.242 | 0.326 |
| Irregular | 586(18.7) | 171(15.5) |  |  |
| Generally regular | 2165(69.2) | 613(69.0) |  |  |
| Regular | 376(12.0) | 126(15.5) |  |  |

**Supplementary Table 2.** Content and abbreviation of items for IAT.

| Node | Content | Abbreviation |
| --- | --- | --- |
| IAT1 | How often do you find that you stay online longer than you intended? | Stay online longer than intended |
| IAT2 | How often do you neglect household chores to spend more time online? | Neglect household chores |
| IAT3 | How often do you prefer the excitement of the internet to intimacy with your partner? | Prefer contacting Internet to partner |
| IAT4 | How often do you form new relationships with fellow online users? | Form new relationships online |
| IAT5 | How often do others in your life complain to you about the amount of time you spend online? | Complained about being on the web for very long |
| IAT6 | ow often do your grades or school work suffer because of the amount of time you spend online? | Grades or school work suffer |
| IAT7 | How often do you check your email before something else that you need to do? | Put the Internet before anything else |
| IAT8 | How often does your job performance or productivity suffer because of the internet? | Job performance or productivity suffer |
| IAT9 | How often do you become defensive or secretive when anyone asks you what you do online? | Alert when asked about online |
| IAT10 | How often do you block out disturbing thoughts about your life with soothing thoughts of the internet? | Block out life troubles with the Internet |
| IAT11 | How often do you find yourself anticipating when you will go online again? | Anticipate when going online again |
| IAT12 | How often do you fear that life without the internet would be boring, empty, and joyless? | Life is boring without the Internet |
| IAT13 | How often do you snap, yell, or act annoyed if someone bothers you while you are online? | Annoyed if being bothered while online |
| IAT14 | How often do you lose sleep due to being online? | Lose sleep due to the Internet |
| IAT15 | How often do you feel preoccupied with the internet when offline, or fantasize about being online? | Fantasize about the Internet when off-line |
| IAT16 | How often do you find yourself saying “just a few more minutes” when online? | Lack of self-control when being online |
| IAT17 | How often do you try to cut down the amount of time you spend online and fail? | Fail to cut down the time spend online |
| IAT18 | How often do you try to hide how long you have been online? | Hide the duration time online |
| IAT19 | How often do you choose to spend more time online over going out with others? | Prefer being online to going out with others |
| IAT20 | How often do you feel depressed, moody, or nervous when you are offline, which goes away once you are back online? | Feel in terrible emotion when off-line |

**Note:** IAT, Internet addiction test; IAT1-IAT20 represents 20 items of IAT.

**Supplementary Table 3.** GEE analysis of factors associated with internet addiction severity.

| Variable | β Coefficient | 95% CI | *P* |
| --- | --- | --- | --- |
| **LPA Profile (Ref: Regular Users)** |  |  |  |
| Moderate Users | 8.52 | 6.89, 10.15 | < 0.001 |
| Addicted Users | 21.73 | 19.24, 24.22 | < 0.001 |
| **Clinical Scales (Continuous)** |  |  |  |
| Insomnia Severity Index (ISI) | 0.45 | 0.32, 0.58 | < 0.001 |
| Patient Health Questionnaire-9 (PHQ-9) | 0.68 | 0.51, 0.85 | < 0.001 |
| Suicidal Behaviors Questionnaire-Revised (SBQ-R) | 0.92 | 0.75, 1.09 | < 0.001 |

**Supplementary Fig. 1.** Heatmap of Spearman correlation coefficients among Internet Addiction Test (IAT) scores, Insomnia Severity Index (ISI) scores, Patient Health Questionnaire‑9 (PHQ‑9) scores, and Suicidal Behaviors Questionnaire‑Revised (SBQ‑R) scores (n = 3,127).

**Supplementary Fig. 2.** Trend of model fit indices (AIC, BIC, adjusted BIC) across latent profile models with 2 to 5 classes. Lower values indicate better model fit, with consideration of parsimony and interpretability in final model selection.

**Supplementary Fig. 3.** Mean score profiles of Internet Addiction Test (IAT) items for latent profile models with 2, 3, 4, and 5 classes.

**Supplementary Fig. 4.** Results of bootstrapped difference test for nodes of internet addiction network. The black grid indicates a significant difference between the two corresponding edge weights, and the gray grid indicates no significant difference.

**Supplementary Fig. 5.** Bootstrap analysis results of the edge weights of internet addiction network. The gray area represents the bootstrap 95% confidence interval (CIs), narrower CIs indicate reliable accuracy.

**Supplementary Fig. 6.** Stability of strength by case dropping subset bootstrap of internet addiction network.

**Supplementary Fig. 7.** Centrality indices of the network between internet addiction and mental disorders

**Supplementary Fig. 8.** Results of bootstrapped difference test for strength within the internet addiction-mental disorders network. The black grid indicates a significant difference between the two corresponding edge weights, and the gray grid indicates no significant difference.

**Supplementary Fig. 9.** Results of bootstrapped difference test for bridge strength within the internet addiction-mental disorders network. The black grid indicates a significant difference between the two corresponding edge weights, and the gray grid indicates no significant difference.

**Supplementary Fig. 10.** Bootstrap analysis results of the edge weights within the internet addiction-mental disorders network. The gray area represents the bootstrap 95% confidence interval (CIs), narrower CIs indicate reliable accuracy.

**Supplementary Fig. 11.** Stability of centrality indices by case dropping subset bootstrap of internet addiction-mental disorders network.

**Supplementary Fig. 12.** Stability of bridge centrality indices by case dropping subset bootstrap of internet addiction-mental disorders network.

**# network analysis Script**

library(readxl)

library(qgraph)

library(bootnet)

library(foreign)

library(Hmisc)

library(networktools)

library(haven)

library(Rcpp)

library(corpcor)

library(EstimateGroupNetwork)

library(mgm)

library(ggplot2)

library(boot)

library(NetworkComparisonTest)

#A

data1<-read.csv("Class3.csv",header = TRUE)

colnames(data1)

c1=cor_auto(data1)

qgraph<-qgraph(c1)

qgraph<-qgraph(cor_auto(data1))

ICU26groups<-c("PHQ-9","PHQ-9","PHQ-9","PHQ-9","PHQ-9","PHQ-9","PHQ-9","PHQ-9","PHQ-9",

"SBQ-R","SBQ-R","SBQ-R","SBQ-R",

"ISI","ISI","ISI","ISI","ISI","ISI","ISI",

"IAT","IAT","IAT","IAT","IAT","IAT")

labels<-colnames(data1)

Names<-c("Low interest or pleasure","Feeling down, hopeless","Trouble sleeping","Tired or little energy","Poor appetite/overeating","Guilt","Trouble concentrating","Moving slowly/restless","Suicidal thoughts",

"History of suicidal thoughts, plans or attempts","Frequency of suicidal ideation in the past year","Communication of intent to die by suicide","Self-reported likelihood of death by suicide",

"Severity of sleep-onset (initial)","Sleep maintenance (middle)","Early morning awakening (terminal) problems","Satisfaction with current sleep pattern","Interference with daily functioning","Noticeability of impairment attributed to the sleep problem","Level of distress caused by the sleep problem",

"Excessive use","Neglect of social life","Lack of control","Neglect of work","Preoccupation","Salience")

Network_glasso<-estimateNetwork(data1,default = "EBICglasso",corMethod="cor_auto")

pdf("Class3IAT_20item.pdf",width = 18,height = 16)

plot(Network_glasso,layout="spring",groups=ICU20groups,

vsize=6,legend=TRUE,borders=TRUE,

palette="colorblind",

legend.cex=0.5,label.cex=1,

height=18,width=16,

labels=labels,

nodeNames=Names,mar=c(4,1,2,2))

dev.off()

centrality_auto(Network_glasso)

centralityPlot(Network_glasso,include = c("Strength","Closeness","Betweenness"))

centralityPlot(Network_glasso,orderBy = "Strength",scale = c("z-scores"),include = c("Betweenness","Closeness","Strength"))

Results1<-bootnet(Network_glasso,statistics = c("Strength","Betweenness","Closeness","edge"),nBoots = 1000,nCores = 8)

plot(Results1,"Strength",plot = "difference")

plot(Results1,"edge",plot = "difference",onlyNonZero = TRUE,order = "sample")

plot(Results1,labels = FALSE,order = "sample")

Results2<-bootnet(Network_glasso,statistics = c("Strength","Betweenness","Closeness"),nBoots = 1000,nCores = 8,type = "case")

plot(Results2,statistics = c("Strength"))

corStability(Results2)

#B

data1<-read.csv("Class3.csv",header = TRUE)

attach(data1)

short.names<-c("P1","P2","P3","P4","P5","P6","P7","P8","P9",

"S1","S2","S3","S4",

"I1","I2","I3","I4","I5","I6","I7",

"A1","A2","A3","A4","A5","A6")

long.names<-c("Low interest or pleasure","Feeling down, hopeless","Trouble sleeping","Tired or little energy","Poor appetite/overeating","Guilt","Trouble concentrating","Moving slowly/restless","Suicidal thoughts",

"History of suicidal thoughts, plans or attempts","Frequency of suicidal ideation in the past year","Communication of intent to die by suicide","Self-reported likelihood of death by suicide",

"Severity of sleep-onset (initial)","Sleep maintenance (middle)","Early morning awakening (terminal) problems","Satisfaction with current sleep pattern","Interference with daily functioning","Noticeability of impairment attributed to the sleep problem","Level of distress caused by the sleep problem",

"Excessive use","Neglect of social life","Lack of control","Neglect of work","Preoccupation","Salience")

groups<-list("PHQ-9"=c(1,2,3,4,5,6,7,8,9),

"SBQ-R"=c(10,11,12,13),

"ISI"=c(14,15,16,17,18,19,20),

"IAT"=c(21,22,23,24,25,26))

colour<-list("PHQ-9"=c(1,2,3,4,5,6,7,8,9),

"SBQ-R"=c(10,11,12,13),

"ISI"=c(14,15,16,17,18,19,20),

"IAT"=c(21,22,23,24,25,26))

colnames(data1)<-short.names

View(data1)

ggm<-estimateNetwork(data1,default = "EBICglasso",verbose = FALSE)

getWmat(ggm)

adj.ggm<-getWmat(ggm)

round(adj.ggm,3)

write.csv(adj.ggm,"Cor_Class3.csv")

Network2<-qgraph(adj.ggm,layout="spring",

labels=short.names,nodeNames=long.names,

groups=colour,

cut=0,vsize=6,border.width=1.5,

theme="colorblind",legend.cex=.40)

pdf("Network_Class3.pdf",width = 16,height = 14)

qgraph(Network2,layout="spring",

labels=short.names,nodeNames=long.names,

groups=colour,

cut=0,vsize=6,border.width=1.5,

theme="colorblind",negDashed=TRUE,legend.cex=.40)

dev.off()

centrality<-centrality_auto(Network2,weighted = TRUE,signed = TRUE)

nc<-centrality$node.centrality

nc

SPL<-centrality$ShortestPathLengths

SPL

pdf("Strength3.pdf",width = 14,height = 12)

qgraph::centralityPlot(Network2,include = c("Closeness","Betweenness","Strength"),scale = "raw")

qgraph::centralityPlot(Network2,orderBy = "Closeness", include = c("Closeness","Betweenness","Strength"),scale = "z-scores")

dev.off()

boot.edges<-bootnet(ggm,nBoots = 1000,nCores = 8)

pdf("edge_Class3.pdf",width = 20,height = 18)

plot(boot.edges,"edge",plot="difference",onlyNonZero=TRUE,order="sample")

dev.off()

A<-adj.ggm

B<-bridge(A,communities = groups,directed = FALSE)

pdf("Bridge_Class3.pdf",width = 14,height = 12)

plot(B, order = "value", zscore = TRUE, include = c("Bridge Strength", "Bridge Betweenness","Bridge Closeness"))

B

dev.off()

bootPro_1<-bootnet(Network_glasso,default = c("EBICglasso"),statistics = c("bridgeStrength"),nBoots = 1000,nCores = 8,communities = groups)

pdf("bridgeStrength_Class3.pdf",width = 20,height = 18)

plot(bootPro_1,"bridgeStrength",plot = "difference",order = "sample")

dev.off()

bootPro_2<-bootnet(Network_glasso,default = c("EBICglasso"),statistics = c("bridgeStrength","bridgeBetweenness","bridgeCloseness"),nBoots = 1000,type = "case", nCores = 8,communities = groups)

plot(bootPro_2,c("bridgeStrength","bridgeBetweenness","bridgeCloseness"))

\#

data1<-read.csv("2.18class3_gender1.csv",header = TRUE)

data2<-read.csv("2.18class3_gender2.csv",header = TRUE)

data3<-read.csv("Class3.csv",header = TRUE)

Network1<-bootnet::estimateNetwork(data1,default = "EBICglasso",corMethod="cor_auto")

Network2<-bootnet::estimateNetwork(data2,default = "EBICglasso",corMethod="cor_auto")

Network3<-bootnet::estimateNetwork(data3,default = "EBICglasso",corMethod="cor_auto")

groups<-c("P1","P2","P3","P4","P5","P6","P7","P8","P9",

"S1","S2","S3","S4",

"I1","I2","I3","I4","I5","I6","I7",

"A1","A2","A3","A4","A5","A6")

Labels<-colnames(data1)

Names<-c("Low interest or pleasure","Feeling down, hopeless","Trouble sleeping","Tired or little energy","Poor appetite/overeating","Guilt","Trouble concentrating","Moving slowly/restless","Suicidal thoughts",

"History of suicidal thoughts, plans or attempts","Frequency of suicidal ideation in the past year","Communication of intent to die by suicide","Self-reported likelihood of death by suicide",

"Severity of sleep-onset (initial)","Sleep maintenance (middle)","Early morning awakening (terminal) problems","Satisfaction with current sleep pattern","Interference with daily functioning","Noticeability of impairment attributed to the sleep problem","Level of distress caused by the sleep problem",

"Excessive use","Neglect of social life","Lack of control","Neglect of work","Preoccupation","Salience")

L<-averageLayout(Network1,Network2)

pdf("Fig_sibling11-3.pdf",width = 12,height = 10)

par(mfrow=c(2,2))

Network1G<-plot(Network1,layout= L,title="Class1",groups=groups,vsize=8,legend=FALSE,borders=TRUE,palette="colorblind",label.cex=1)

Network2G<-plot(Network2,layout= L,title="Class2",groups=groups,vsize=8,legend=FALSE,borders=TRUE,palette="colorblind",label.cex=1)

Network3G<-plot(Network2,layout= L,title="Class3",groups=groups,vsize=8,legend=FALSE,borders=TRUE,palette="colorblind",label.cex=1)

dev.off()

compare_12<-NCT(data1,data2,binary.data = FALSE,paired = FALSE,test.edges = TRUE,edges = 'all',progressbar=TRUE,test.centrality=TRUE,centrality="strength")

View(compare_13)

summary(compare_12)

#validation for Table 1

\# --- Global sample size ---

total_n <- 3127

\# --- Helper function to validate counts and percentages ---

validate_section <- function(section_name, df) {

cat("\n--- Checking:", section_name, "---\n")

\# Check if row count sums to total_n

sum_counts <- sum(df$Count)

if(sum_counts != total_n){

cat(" Row total mismatch! Sum =", sum_counts, "Expected =", total_n, "\n")

} else {

cat("✔ Row total matches sample size.\n")

}

\# Check if calculated percentages match reported (allowing small rounding error ±0.1)

calc_pct <- round(df$Count / total_n * 100, 1)

pct_diff <- abs(calc_pct - df$Percent) > 0.1

if(any(pct_diff)){

cat("❌ Percentage mismatch detected in rows:\n")

print(df[pct_diff, ])

cat("Calculated percentages:", calc_pct[pct_diff], "\n")

} else {

cat("✔ Percentages match reported values.\n")

}

}

\# === 1. Gender ===

gender_df <- data.frame(

Category = c("Male", "Female"),

Count = c(1410, 1717),

Percent = c(45.1, 54.9)

)

validate_section("Gender", gender_df)

\# === 2. Race ===

race_df <- data.frame(

Category = c("Han", "Other"),

Count = c(2989, 138),

Percent = c(95.6, 4.4)

)

validate_section("Race", race_df)

\# === 3. Residence ===

residence_df <- data.frame(

Category = c("Urban", "Rural"),

Count = c(1042, 2085),

Percent = c(33.3, 66.7)

)

validate_section("Residence", residence_df)

\# === 4. Family sibling status ===

sibling_df <- data.frame(

Category = c("Only child", "Non-only child"),

Count = c(929, 2198),

Percent = c(29.7, 70.3)

)

validate_section("Family sibling status", sibling_df)

\# === 5. BMI ===

bmi_df <- data.frame(

Category = c("Low weight", "Normal", "Overweight/obesity"),

Count = c(471, 1945, 711),

Percent = c(15.1, 62.2, 22.7)

)

validate_section("BMI", bmi_df)

\# === 6. Smoking status ===

smoking_df <- data.frame(

Category = c("Yes", "No"),

Count = c(225, 2902),

Percent = c(7.2, 92.8)

)

validate_section("Smoking status", smoking_df)

\# === 7. Alcohol status ===

alcohol_df <- data.frame(

Category = c("Yes", "No"),

Count = c(618, 2509),

Percent = c(19.8, 80.2)

)

validate_section("Alcohol status", alcohol_df)

\# === 8. Physical activity ===

physical_df <- data.frame(

Category = c("Low", "Moderate", "High"),

Count = c(171, 2037, 919),

Percent = c(5.5, 65.1, 29.4)

)

validate_section("Physical activity", physical_df)

\# === 9. Regularity of meals ===

meals_df <- data.frame(

Category = c("Irregular", "Generally regular", "Regular"),

Count = c(586, 2165, 376),

Percent = c(18.7, 69.2, 12.0)

)

validate_section("Regularity of meals", meals_df)

cat("\n Validation completed.\n")
